# Correlation of Depression and Anxiety With Rheumatoid Arthritis

**DOI:** 10.7759/cureus.23137

**Published:** 2022-03-14

**Authors:** Emilia E Jones Amaowei, Sana Anwar, Kumudhavalli Kavanoor Sridhar, Khadeja Shabbir, Ehtesham H Mohammed, Abdul Rasheed Bahar, Abdul Subhan Talpur, Sadaf Bhat, Shumaila Zafar, Laila Tul Qadar

**Affiliations:** 1 Clinical Affairs, Dexcom Inc, San Diego, USA; 2 Medicine, St Christopher Iba Mar Diop College of Medicine, Dakar, SEN; 3 Internal Medicine, Lugansk State Medical University, Luhansk, UKR; 4 Medical Microbiology, Rehoboth Diagnostics Centre, Chennai, IND; 5 Medical Microbiology, The Tamilnadu Dr.M.G.R Medical University, Chennai, IND; 6 Internal Medicine, Saveetha Medical College, Chennai, IND; 7 Medicine, Mount Sinai Hospital, Toronto, CAN; 8 Internal Medicine, Hacettepe University School of Medicine, Ankara, TUR; 9 Medicine, Liaquat University of Medical and Health Sciences, Jamshoro, PAK; 10 Internal Medicine, Liaquat University of Medical and Health Sciences, Jamshoro, PAK; 11 Internal Medicine, Gynaecology and Obstetrics, Mayo Hospital, Lahore, PAK; 12 Internal Medicine, Civil Hospital Karachi, Karachi, PAK

**Keywords:** rheumatoid arthritis, pain, inflammation, hads scale, depression, arthritis, anxiety

## Abstract

Background

Psychiatric comorbidity with a chronic disease is linked with poor patient outcomes. Therefore, the current research assessed the correlation of rheumatoid arthritis (RA) with depression and anxiety disorders.

Methodology

A prospective observational study was undertaken at a public sector hospital between December 2020 to June 2021. All individuals who presented with rheumatoid arthritis were included in the study. A healthy cohort acted as the control group. Depression and anxiety were assessed using the Hamilton depression rating scale (HDRS) and the Hamilton anxiety rating scale (HAM-A), respectively. The patients were inquired about their gender, age, and duration of RA. Further stratification was done using the Chi-squared test. A p-value of <0.05 was decided as the cut-off for significance. All data from the patients were collected in a predefined pro forma.

Results

A total of 169 patients with RA and 85 healthy controls were enrolled in the study. The mean depression score among patients with RA was 19.65 ± 1.44 versus 14.4 ± 1.31 in the control group (p<0.001). Moreover, the mean anxiety score in patients with RA was 19.44 ± 2.4. About 71% of patients with RA were diagnosed with psychiatric issues, while only 7.1% of individuals in the control group had either depression or anxiety (p<0.0001). Furthermore, it was found that the majority of the patients with RA had depression with a frequency of 70 (58.3%), while only six participants in the control group had depression. None of the participants had moderate or severe depression. However, 16 (69.6%) patients with RA had major anxiety issues. In 27 patients, mixed anxiety-depression disease was diagnosed. Out of these, 23 (85.2%) had the depression-dominant mixed disorder.

Conclusion

The present study highlighted the alarming incidence of depression and anxiety among patients with RA. Furthermore, it also indicated the relationship between severity of psychiatric comorbidity with chronic rheumatoid arthritis in our population. Further large-scale studies are needed to ascertain the demographic confounders that may help predict psychiatric disorders among patients with RA.

## Introduction

Current insights into psychiatry reveal that inflammation plays a significant role in the pathogenesis of psychiatric diseases such as depression and anxiety. Depression is correlated with several physical illnesses including COVID-19, cardiovascular diseases, and long-standing ailments such as cancer [1,2]. Recent advances have also noted that inflammatory conditions and infectious states can lead to psychiatric morbidity in patients [3].

Characterized by chronic inflammation, rheumatoid arthritis (RA) most commonly affects the joints and has a prevalence of 0.5% to 1% [4]. Many clinical studies noted that depression was a common feature in patients suffering from RA and was seen in about 13% to 20% of the patients [5-7]. However, the incidence rate has been inconsistently reported in the literature [6]. 

Rheumatoid arthritis is frequently linked with poor patient outcomes, causing severe pain, lethargy, disability, and low compliance to treatment regimens [6,7]. A systematic review conducted in the year 2013 [8] found that the co-occurrence of depression in patients suffering from RA was associated with the worsening of pain and a reduction in the positive effects of treatment.

A study by Matcham et al. concluded that there existed a significant association between depression and the severity of RA progression. It was noted that the greater the severity of depression at baseline, the greater was the disease activity found at follow-up after a year [9]. Another study revealed that depression if found at baseline in patients suffering from RA, was associated with a reduction of the effects of prednisolone by 50% as compared to patients of RA with no symptoms of depression [10].

Due to the scarcity of local literature, the current study was conducted to evaluate the burden of anxiety as well as depression among cohorts of RA in the Pakistani population.

## Materials and methods

A prospective observational study was undertaken at a public sector hospital between December 2020 to June 2021. Ethical permission from the institutional review board (IRB #LUMHS/IRB/88495) was obtained before the initiation of the study. Informed written consent was obtained from the participants. A non-probability convenience sampling technique was employed for the recruitment of patients. 

By keeping the prevalence of depression among patients with RA as 15.29%, a margin of error as 5.42%, and a confidence level of 95%, a sample size of 169 was obtained [6]. All patients who gave consent and aged between 18 to 70 years, irrespective of sex, were diagnosed with RA according to the American College of Rheumatology (ACR), with a duration of disease > two years were included in the study. Patients with carcinoma, autoimmune disease, or those with osteomalacia, osteoarthritis, and any other bone disease were excluded. 

All patients were assessed for sociodemographic parameters including gender, age, and the number of years since RA diagnosis since these were potential confounders found in the literature. Depression and anxiety were evaluated with the aid of the Urdu version of the Hamilton depression rating scale (HDRS) and the Hamilton anxiety rating scale, consecutively [11,12]. Both HDRS and Hamilton anxiety rating scales are reliable and valid instruments for the evaluation of depression and anxiety. The HDRS reported satisfactory inter-rater and test-retest reliability [11]. Both instruments were used to assess the severity of depression and anxiety among patients with RA and the controls. Each item on the scales is scored from a 0 (not present) to 4 (severe). The questionnaires were administered by a senior resident who was blinded to the objective of the study to prevent bias.

Data were analyzed using Statistical Package for the Social Sciences (SPSS) version 24 (IBM Corp., Armonk, NY, US). Mean (SD) was determined for age, height, weight, BMI, duration of RA, education level, number of children, and number of family members. For all categorical variables, including gender, the severity of anxiety and depression were presented as proportions. A Chi-squared test was applied to evaluate the correlation between mental health issues and the presence of RA. Furthermore, patients were stratified as per their sociodemographic and clinical parameters. A probability level of below 0.05 was considered to be the cut-off for statistical significance. 

## Results

About 169 patients with RA and 85 healthy controls were enrolled in the study. The mean ± SD age of the study group and the control group was 54.3 ± 10.4 and 51.8 ± 9.1 years, consecutively (p=0.22). The mean diagnosis duration of RA was 5 ± 3.1 years. There was a major female predominance with 142 (84%) females with RA (Table 1). 

**Table 1:** Sociodemographic and clinical parameters in patients with rheumatoid arthritis versus control ANA (IFA): Antinuclear antibodies (indirect immunofluorescence assay), RA: Rheumatoid arthritis

Characteristics	RA (n=169)	Control (n=85)	p-value
Gender			0.407
Male	27 (16%)	19 (22.4%)	
Female	142 (84%)	66 (77.6%)	
Rheumatoid factor titer (U)	177.3 ± 186	10.3 ± 59	<0.0001
Rheumatoid factor positivity (%)			<0.0001
Positive	142 (84%)	4 (4.7%)	
Negative	27 (16%)	81 (95.3%)	
ANA (IFA) positivity (%)			0.002
Positive	61 (36.1%)	8 (9.4%)	
Negative	108 (63.9%)	77 (90.6%)	

The mean depression score among patients with RA was 19.65 ± 1.44 versus 14.4 ± 1.31 in the control group (p<0.001). Moreover, the mean anxiety score in patients with RA was 19.44 ± 2.4. As illustrated in Table 2, about 71% of patients with RA were diagnosed with psychiatric issues while only 7.1% of individuals in the control group had either depression or anxiety (p<0.0001).

**Table 2:** Correlation between severity of psychiatric illness with rheumatoid arthritis RA: Rheumatoid arthritis

	RA (n=169)	Control (n=85)	p-value
Psychiatric evaluation			<0.0001
Normal psychiatric examination	49 (29%)	79 (92.9%)	
Incidence of psychiatric issue	120 (71%)	6 (7.1%)	
Type of psychiatric issue			0.126
Depressive disorder	70 (58.3%)	6 (100%)	
Anxiety disorder	23 (19.2%)	-	
Mixed anxiety-depressive disorder	27 (22.5%)	-	
Severity of depressive disorder			<0.0001
Mild depression	70 (100%)	6 (100%)	
Moderate-severe depression	-	-	
Occurrence of anxiety			0.009
Mild anxiety	7 (30.4%)	-	
Severe anxiety	16 (69.6%)	-	
Both anxiety-depression			0.004
Dominance by depressive symptoms	23 (85.2%)	-	
Dominance by anxiety symptoms	4 (14.8%)	-	

Furthermore, it was found that the majority of the patients with RA had depression with a frequency of 70 (58.3%), while only six participants in the control group had depression. None of the participants had moderate or severe depression. However, 16 (69.6%) patients with RA had major anxiety issues. In 27 patients, mixed anxiety-depression disease was diagnosed. Out of these, 23 (85.2%) had the depression-dominant mixed disorder. 

## Discussion

Rheumatoid arthritis (RA) is characterized as a debilitating systemic inflammatory disorder that causes physical and psychological impairment [13]. Depression and anxiety very commonly co-occur in patients suffering from RA [10]. Psychological disorders in association with RA results in poor disease prognosis [14]. The impact of depression on RA is much greater than its burden of mental illness itself [6,7].

The present study found a significantly higher percentage of depressive episodes and anxiousness among those with RA than healthy individuals without the debilitating disease. In similar studies, the propensity of depression in patients of RA lay between the range of 13% to 42% [15-18], which was four times that seen among the healthy population. The wide range of prevalence of depression may have occurred because of the various ways in which the symptoms of depression were assessed and evaluated [19]. The patient’s race could also be an independent factor that increases the likelihood of depressive symptoms in those individuals with RA. The incidence of depression among Asians suffering from RA was much lower [20], while Hispanics suffering from RA reported a much higher prevalence of depression [21]. The difference in incidence rates between different ethnicities could be attributed to a myriad of factors such as genetics, environmental, cultural values, lifestyle, and dietary habits. 

Numerous cross-sectional studies have reported a significant association between the progression of RA and depression [20-22]. This association could be secondary to psychological factors, or there may be unknown factors driving this association. Wright et al. comprehensively reviewed the relationship between depression and inflammatory conditions and diseases and found that COVID-19 infection is significantly associated with psychiatric comorbidity [23]. Depression may be an indirect effect of RA, which may be linked with the burden of RA symptoms or the progressive and unpredictable nature of RA, which makes patients more susceptible to developing the symptoms of depression [24]. 

The co-occurrence of depression in individuals with RA warrants its regular screening and monitoring [25]. The occurrence of anxiety disorder in individuals with RA is quite high as compared to the general healthy population, as evidenced by the current study. Regardless, patients remain undiagnosed as the symptoms of both depression and anxiety are quite similar to sleep disturbances, stress, or fatigue, which makes differentiating these diseases much harder.

Doctors commonly skip the evaluation of a patient’s mental state during routine checkups for RA, often due to the scarcity of resources, limited time, or the idea that psychological evaluation should be done by a psychiatrist. Patients of RA suffering from undiagnosed psychiatric comorbidity have a much harder time understanding the disease, making it all the more difficult for them to adhere to treatments and cope with the complexities of the disease [26]. Patients of RA with depression or anxiety often find it difficult to make decisions regarding their health and fail to follow treatment protocols. Therefore, it is necessary to screen patients with RA for probable depression or anxiety. 

There were some limitations that the authors of the present study had to face during data acquisition. Firstly, this was a single-centered study thus, we could not include a more diversified sample size. Furthermore, due to the small sample size, the findings of the study cannot be generalized to a larger population. It is recommended that a more comprehensive approach should be targeted in the future. A more diversified sample population would be able to provide a more comprehensive analysis of the bidirectional relationship between psychiatric ailments and endocrinological illnesses. 

## Conclusions

The present study highlighted the alarming incidence of psychiatric disorders among individuals with rheumatoid arthritis. Furthermore, it also indicated the relationship between severity of psychiatric comorbidity with chronic RA in our population. We recommend that patients should be screened for depression and anxiety regularly to avoid further complications associated with rheumatoid arthritis. Moreover, multi-scale studies are needed to ascertain the demographic factors correlated with psychiatric comorbidity among patients with long-standing RA. 
